# “Forms” of water mites (Acari: Hydrachnidia): intraspecific variation or valid species?

**DOI:** 10.1002/ece3.704

**Published:** 2013-08-28

**Authors:** Jeanette Stålstedt, Johannes Bergsten, Fredrik Ronquist

**Affiliations:** 1 Zoology Department, Swedish Museum of Natural HistoryBox 50007, SE-104 05, Stockholm, Sweden; 2 Department of Biodiversity Informatics, Swedish Museum of Natural HistoryBox 50007, SE-104 05, Stockholm, Sweden

**Keywords:** Bayesian analysis, cryptic species, DNA barcoding, GMYC model, principal component analysis, species delimitation

## Abstract

In many groups of organisms, especially in the older literature, it has been common practice to recognize sympatrically occurring phenotypic variants of a species as “forms”. However, what these forms really represent often remains unclear, especially in poorly studied groups. With new algorithms for DNA-based species delimitation, the status of forms can be explicitly tested with molecular data. In this study, we test a number of what is now recognized as valid species of water mites (Hydrachnidia), but have in the past been treated as forms sympatrically occurring with their nominate species. We also test a form without prior taxonomical status, using DNA and morphometrics. The barcoding fragment of COI, nuclear 28S and quantitative analyses of morphological data were used to test whether these taxa merit species status, as suggested by several taxonomists. Our results confirm valid species. Genetic distances between the form and nominate species (*Piona dispersa* and *Piona variabilis,* COI 11%), as well as likelihood ratio tests under the general mixed-Yule coalescent model, supported that these are separately evolving lineages as defined by the unified species concept. In addition, they can be diagnosed with morphological characters. The study also reveals that some taxa genetically represent more than one species. We propose that *P. dispersa* are recognized as valid taxa at the species level. *Unionicola minor* (which may consist of several species), *Piona stjordalensis*, *P. imminuta* s. lat., and *P. rotundoides* are confirmed as species using this model. The results also imply that future studies of other water mite species complexes are likely to reveal many more genetically and morphologically distinct species.

## Introduction

A sound taxonomic foundation is fundamental for all biological sciences from ecology and conservation biology to proteomics and genomics (Wheeler et al. 2004; Wilson 2004). The circumscription and naming of taxa enable the quantification of meaningful units as well as reproducibility within and between scientific studies, the very cornerstone of science. However, species show variable degrees of intraspecific variation, which may be geographically structured, and species delimitation is not always straightforward (Sites and Marshall 2003, 2004). With a vast and complex literature on different species concepts (e.g., Ruse 1969; Nixon and Wheeler 1990; Mayden 1997; Wheeler and Meier 2000), it is encouraging that a consensus view now seems to be emerging, according to which species are seen as separately evolving metapopulation lineages (de Queiroz 2007). Adhering to this “unified species concept” enables more straightforward tests of the validity of species as well as of infrasubspecific taxa. Morphological variants labeled as “forms”, “varieties”, or “ecomorphs” have been described in numerous taxa, both in the past and more recently (Snyder and Hansen 1940; Askew 1970; McLean and Kanner 2005; Mateos 2008). However, what these labels really refer to often remain unclear, undefined or, untested with quantitative data. The international code of zoological nomenclature (ICZN 1999) establishes that infrasubspecific names of the type “var.” and “form” are valid as subspecific names only, if described before 1961 and the author did not explicitly intend them to be of infrasubspecific rank. Here, we leave the debate on subspecies aside, because it is only relevant for allopatric or parapatric distributions (circular range overlap excepted; Wilson and Brown 1953; Starrett 1958; Wilson 1994). Names that refer to sympatrically occurring phenotypic forms or varieties can be explicitly tested using recent advances in applying molecular data and statistical analyses (Sites and Marshall 2003, 2004; Pons et al. 2006; Fontaneto et al. 2007; Knowles and Carstens 2007; Rosenberg 2007; Rodrigo et al. 2008).

Based on the ideas of the unified species concept, there are multiple relevant lines of evidence of speciation, all of which are found in previous species concepts, but as part of the definition (de Queiroz 2007). Examples include the cessation of geneflow, phenetical distinctiveness, diagnosability, ecological niche differentiation, and reciprocal monophyly and several recent methods have been developed to quantitatively test the evidence in favour of, or against, speciation. The general mixed Yule coalescence method (GMYC) (Pons et al. 2006; Fontaneto et al. 2007) provides a quantitative way of circumscribing species without any prior knowledge using single-locus DNA. The method only delimits reciprocally monophyletic species, hence all recognized species under the GMYC model satisfy at least that nonabsolute, but indicative criterion. Specifically, GMYC combines the coalescent process model for populations with the Yule speciation model for species to find the maximum likelihood threshold solution of an ultrametric gene tree. It separates branches that likely represent separate species from branches that are better modeled as within-species coalescents. Rosenberg (2007) and Rodrigo et al. (2008) developed different tests, but aimed at testing the same null hypothesis: could the observed pattern be derived by chance from a single- panmictic population? In Rosenberg's (2007) test, the pattern observed is two reciprocally monophyletic clades and the sample size of each clade determines the probability of observing the pattern under a single-panmictic population. Rodrigo et al.'s (2008) test instead focus on the branch length ratio of the assumed species ingroup node to the tips and the ingroup node to the immediate ancestral node. This is basically a quantitative measure of the “distinctiveness of clusters” often referred to visually on NJ-trees in DNA barcoding studies (Hogg and Hebert 2004; Koch 2010), but is here tested against the probability of seeing the observed ratio under a single-panmictic population. Rejecting the null under both tests imply reduced or absent geneflow between populations and if sympatrically occurring, evidence of species.

Preferably, the circumscription of separately evolving metapopulation lineages should be based on multiple lines of evidence (de Queiroz 2007), why we use quantitative morphological, nuclear, and mitochondrial data for species delimitation. This integrative taxonomic methodology is a powerful tool in resolving taxonomical problems and will in this study on water mites (Hydrachnidia) be applied to already known species (*Unionicola minor* (Soar, 1900)*, Piona stjordalensis* (Thor 1897) [=*curvipes stjørdalensis*]*, P. imminuta* s. lat. (Piersig 1897)*, P. rotundoides* (Thor 1897)) (Biesiadka 1977; Davids and Kouwets 1987; Gerecke 2011), which have in the past been regarded as intraspecific forms to a sympatrically occurring nominate species (*U. minor* in relation to *U. crassipes* (Müller, 1776), *P. stjordalensis* and *P. imminuta* s. lat. both in relation to *Piona coccinea* (Koch, 1836), *P. rotundoides* in relation to *P. pusilla* (Neuman, 1875)) (Viets 1982, 1987). We also test a form presently without accepted species status, synonymous to the nominate species (*P. dispersa* Sokolow 1926 in relation to *P. variabilis* (Koch, 1836)) (Böttger and Ullrich 1974; Gerecke 2011). They can all be found in freshwater habitats in Europe and have a chaotic taxonomical history (Lundblad 1962; Viets 1987; European Water Mite Research 2009). For example, the following taxon names are also involved in the same species complexes, but of debated taxonomic status: *U. crassipes f. octopora* Maglio, 1924, *U. crassipes f. reducta* Lundblad 1924; *U. laurentiana* Crowell and Davids 1979; *U. nearctica* Crowell and Davids 1979; *P. coccinea f. confertipora* Walter, 1927, *P. coccinea f. hankensis* Sokolow, 1931, *Piona coccinea f. recurva* Lundblad 1920; *P. coccinea f. gracilipalpis* Lundblad 1924; the colour variant *P. coccinea f. caesia* Thor, 1925; *P. pusilla f. disjuncta* Viets, 1930, the smaller variant *P. pusilla f. tenera* Lundblad 1925, *P. pusilla f. disparilis* (Koenike, 1895), *P. pusilla f. acutipes* Viets, 1954, *P. pusilla f. rotundiformes* Lundblad, 1938, *P. africana* Viets, 1940, and *P. sudamericana* Viets, 1910) (Lundblad 1920, 1924, 1962; Viets 1982, 1987). Within the species-rich Hydrachnidia, variable sympatrically occurring intraspecific populations have in the past frequently been called forms (Lundblad 1962; Viets 1982, 1987). Despite the large extent of water mite forms currently still unsolved, for example, the problematic *P. nodata* group, there are few molecular studies on cryptic water mite species (but see Edwards and Dimock 1997; Bohonak 1999; Edwards et al. 1999; Bohonak et al. 2004; Ernsting et al. 2006, 2008). This is the first time the status of *Unionicola minor, Piona stjordalensis, P. imminuta* s. lat.*, P. rotundoides,* and *P. dispersa* are tested using molecular data. We apply statistical phylogenetic, species delimitation, and population genetic methods to explicitly test diagnosability, geneflow, monophyly, and phenetic distinctiveness.

## Material and Methods

### Biological material sampled

All included taxa were collected in the years 2007–2008 in Sweden. Specimens identified and extracted were *Unionicola crassipes* (14♀), *U. minor* (12♀), *Piona coccinea* (10♂), *P. stjordalensis* (6♂, 4♀), *P. imminuta* s. lat. (3♂1♀), *P. pusilla* (9♂, 1♀), *P. rotundoides* (3♂, 1♀), *P. variabilis* (9♀), and *P. dispersa* (10♀). *Piona longipalpis* (Krendowskij, 1878) (Pionidae) (10♀) was included as a reference species for comparison since it neither in the past or present contains described forms (Viets 1987) and *Arrenurus suecicus* Lundblad, 1917 (Arrenuridae) (1♂) was used as an outgroup.

**Figure 1:**
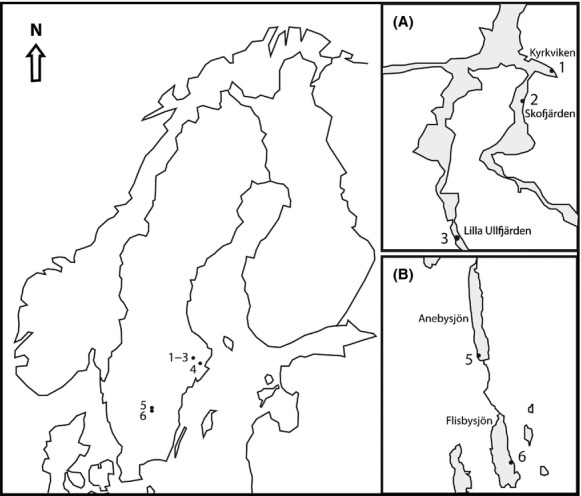
The localities were chosen on the basis of earlier records of targeted nominal species and forms in Sweden (Lundblad 1962, 1968). Material was collected from Lake Mälaren (A; site 1–3), the stream Helgöån (site 4), Lake Anebysjön (B; site 5), and Lake Flisbysjön (B; site 6).

Six localities were chosen on the basis of earlier findings in the provinces of Uppland and Småland (Lundblad 1962, 1968) (Fig. 1, Table 1). The localities included both running and standing water. The examined species were sampled together with its former nominate species in at least one of the sampled localities. *Piona dispersa* coexisted with *P. variabilis* in Lake Mälaren (Fig. 1, Table 1, Appendix 1). Water mites were sampled with a hand net (mesh size 0.5 mm) and sorted in the laboratory. The material was preserved in frozen water (−20°C) until identification and then in ethanol (80%, −20°C). Species were identified with the help of Viets (1936) and Lundblad (1962, 1968). Vouchers and DNA extractions are deposited at the Entomology Department, Swedish Museum of Natural History (NHRS), Stockholm, under the catalogue numbers NHRS-ACAR000000001-94. Images of all vouchers are available on Morphbank (2013; see Appendix 1 for Morphbank accession numbers).

**Table 1:** Localities in the province of Uppland and Småland, Sweden, with coordinates, temperature (air), water depth, and bottom substrate. The habitat of Lilla Ullfjärden and Helgöån (site 3 and 4) lacked dominating plants

	Site (Province *Parish* lake/stream)	Latitude	Longitude	Temp. (°C)	Depth (m)	Bottom substrate
1	**Upl** *Vasslunda* Lake Mälaren, Kyrkviken/Ekhamnsviken	59°43'26.66″N	17°40'49.16″E	8, 15, 20	0–1	*Phragmites,* sand
2	**Upl** *Vasslunda* Lake Mälaren, Skofjärden	59°42'46.17″N	17°38'34.13″E	8, 15, 20	0–1	*Phragmites,* detritus
3	**Upl** *Yttergrans* Lake Mälaren, Lilla Ullfjärden	59°35'25.93″N	17°31'17.46″E	17	0–0.5	Detritus, gravel
4	**Upl** *Össeby* Stream Helgöån	59°36'03.66″N	18°12'45.67″E	18	0–1	Detritus, fine sediment
5	**Sm** *Norra Solberga-Flisby* Lake Anebysjön	57°47'28.84″N	14°48'52.18″E	20	0–1	*Schoenoplectus, Carex,* sand
6	**Sm** *Norra Solberga-Flisby* Lake Flisbysjön	57°44'39.57″N	14°50'51.64″E	20	0–0.5	*Carex, Typha,* sand

### Molecular analysis

The molecular work was carried out at the Molecular Systematics Laboratory (MSL), Swedish Museum of Natural History. DNA was extracted from the tissue of four legs of each individual. In some cases the whole mite was used, with body fluids removed to avoid contamination. The extraction method followed the tissue protocol of Blood and Tissue Genomic Mini kit (Viogene, Taipei, Taiwan). A few individuals were extracted using GeneMole (Mole Genetics, Lysaker, Norway), QIAamp® DNA Mini Kit (Qiagen), or QIAamp® DNA Micro Kit (Qiagen, Hilden, Germany). The protocols were modified by increasing the time of lysis with 4–5 h and using only 50 μL × 2 AE/TE solution in the elution step.

The universal primers LCO 1490-forward 5'-GGTCAACAAATCATAAAGATATTGG-3' and HCO 2198-reverse 5'-TAAACTTCAGGGTGACCAAAAAATCA-3' (Folmer et al. 1994) were used to amplify the 5' fragment of cytochrome c oxidase subunit I (COI) (658 bp). All amplification reactions were done in a total volume of 25 μL containing 3–5 μL DNA, 0.5 μL of each primer (10 μmol/L), and DNA-grade water, using PCR beads (illustra™ Hot Start Mix RTG from GE Healthcare, Buckinghamshire, U.K.). Thermal cycling conditions for COI amplification were as follows: 5 min at 94°C, (30 sec at 94°C, 15 sec at 46°C, 30 sec at 72°C) × 40, 10 min at 72°C. PCR products showing low amplification by gel electrophoresis were reamplified with 20 cycles. A fragment of the D2 region of 28S rDNA (28S) was amplified using primers D2F-forward 5'-AGTCGTGTTGCTTGATAGTGCAG-3' and D2R-reverse 5'-TTGGTCCGTGTTTCAAGACGGG-3' (Campbell et al. 1993; Goolsby et al. 2006). Amplification of 28S was accomplished by 3 min at 94°C, (30 sec at 94°C, 30 sec at 55°C, 30 sec at 72°C) × 42, 10 min at 72°C. PCR products showing low amplification by gel electrophoresis were reamplified with 30 cycles.

PCR products were purified using ExoSAP (Fermentas, Vilnius, Lithuania) for 30 min at 37°C and 15 min at 80°C. Gene regions were sequenced with the same primers as in the PCR using the ABI BigDye™ Terminator ver. 3.1 Cycle Sequencing Kit (Applied Biosystems, Foster city, CA). Each sequencing reaction ran for 1 min at 96°C, (30 sec at 96°C, 15 sec at 50°C, 4 min at 60°C) × 25. Sequencing reactions were purified using the DyeEx 96 kit (Qiagen) and cycle sequencing reactions were run on an ABI 3130xl Genetic Analyzer (Applied Biosystems). Raw sequence data and contigs were viewed and assembled using the Pregap4 and Gap4 modules of the Staden package 1.6.0 (Staden et al. 1998). Primer sequences were removed from the beginning and end of each sequence. The 28S sequences were aligned using the FFT-INS-I strategy in MAFFT v. 6 (Katoh et al. 2005), which resulted in an alignment length of 734 bp. The alignment of COI was straightforward as sequences were length invariant. Only sequences with <15% missing data were used, expect for one 28S sequence with 55% missing values (*Piona pusilla* specimen 7). Sequence data of COI and 28S were available for all examined species including *P. dispersa*. However, some specimens were successfully sequenced for only COI (*P. longipalpis* (5 of 10), *P. variabilis* (3 of 9), and *P. dispersa* (5 of 10)) or 28S (*U*. sp D nr *minor* (1 of 1), *P. coccinea* (3 of 10), *P*. *stjordalensis* (1 of 10)*, P*. *rotundoides* (3 of 4)) (Appendix 1). Nucleotide composition statistics, genetic distances, and parsimony informative characters were obtained using MEGA v.4.1 (Tamura et al. 2007). All sequences, trace files, primer sequences, voucher catalogue numbers, and collection data are submitted to BOLD (Ratnasingham and Hebert 2007). In addition, sequences are deposited in Genbank under the accession codes JN034731-JN034895 (Appendix 1). Phylogenetic trees were reconstructed with Bayesian methods using MrBayes v.3.2.1 (Ronquist and Huelsenbeck 2003). Substitution models (GTR + I + Γ) for COI (with 1st + 2nd and 3rd codon position partitioned separately) and 28S were selected with MrModeltest v. 2.3 (Nylander 2004). All parameters except topology and branch lengths were unlinked across partitions. Markov chain Monte Carlo (MCMC) settings were 10 million generations sampled every 500 generations, with the first 25% of samples discarded as burn-in. We ran both COI and 28S separately. COI and 28S analyses had a standard deviation of split frequencies below 0.01 (0.006, 0.004, respectively). A combined COI and 28S analysis was made with the same models as specified above and had an average standard deviation of split frequencies below 0.02 (0.018) after 15 million generations (not shown). Genetic distances within and between species were calculated with a K2P model (Kimura 1980).

To test if *Piona dispersa* and the examined species are likely to be separately evolving lineages and species under the unified species concept, we used GMYC analysis (Pons et al. 2006; Fujisawa and Barraclough 2013), Rosenberg's (2007) test of reciprocal monophyly and Rodrigo et al.'s (2008) test of branch length ratios. These are all tests for single-locus gene trees. We ran three different GMYC analyses: one separate analysis for each genus (*Piona* and *Unionicola*), and a combined analysis with data from both genera (not shown), as the influence of taxon sampling is a concern (Fujisawa and Barraclough 2013). The GMYC analysis requires an ultrametric tree which was inferred with a clock model in MrBayes after identical COI haplotypes had been removed (Fujisawa and Barraclough 2013). As a strict molecular clock could not be rejected over a nonclock model (likelihood ratio test; *P* < 0.05 for all three datasets), we used a strict-clock model to infer the ultrametric gene tree. The MCMC settings were the same as above, with the run length being 10 million generations. The GMYC analysis was performed in R version 3.0.1 with the ‘splits’ package (Ezard et al. 2013; R Core Team 2013). Rosenberg's (2007) and Rodrigo et al.'s (2008) tests were conducted using the COI trees from MrBayes, with the software Genious and the species delimitation plugin described by Masters et al. (2011).

### Morphometric analysis

Prior to examination, body fluids were removed from the specimen by gently puncturing the body with an insect pin. Measurements of the width and length of the body, fourth coxa (in *Unionicola* specimens the fused coxa III and IV), palpal femur (P-II), and palpal tibia (P-IV), were taken with a Leitz Wetzlar Laborlux S microscope and an ocular micrometer. We also measured the dorsal length of the remaining segments of the palp (Table 2). In addition, we counted the sclerotized and unsclerotized genital acetabula of females, and measured the width and length of the tarsus and claw of the males( third leg. All measurements are given in micrometers. Characters of males and females for each genus were analyzed separately in a principal component analyses performed in R version 2.8.1 (R Development Core Team 2008). Specimen 3 of *P. coccinea* and specimen 4 of *P*. *variabilis* were not included in the analysis due to missing values for palp length.

**Table 2:** Length and width (mean values in μm) of bodysize, coxal plate IV (length of III + IV for *Unionicola*), and palp, as well as the number of genital acebula (right and left side) for each species. Length and width (μm) on claw and tarsus (leg segment) of the males( third leg are shown for *Piona coccinea-* and *P. pusilla*-complex. All values were rounded to integers

Species	*N*	Body	Cx-II + IV	Palp (P-1, P-II, P-III, P-IV, P-V)	Genital acetabula mean(SD)	Claw, tarsus
*Unionicola crassipes* ♀	14	1175 × 950	408 × 330	25–210 × 125–90–210 × 62–172	6 (0) + 6 (0)	
*U*. sp. A nr *minor* ♀	5	779 × 681	276 × 207	14–132 × 91–53–123 × 40–90	6 (0) + 6 (0)	
*U*. sp. B nr *minor* ♀	3	943 × 815	324 × 252	17–154 × 94–57–138 × 49–92	6 (0) + 6 (0)	
*U*. sp. C nr *minor* ♀	3	827 × 645	284 × 216	20–157 × 88–52–131 × 44–97	6 (0) + 6 (0)	
*U*. sp. D nr *minor* ♀	1	1071 × 714	324 × 216	20–155 × 80–55–153 × 40–105	6 (0) + 6 (0)	
*Piona coccinea* ♂	10	1345 × 1120	322 × 421	66–287 × 142–125–316 × 74–159	23 (3) + 23 (2)	83 × 73, 169 × 126
*P*. stjordalensis ♂	6	907 × 856	214 × 319	51–252 × 160–114–261 × 79–126	27 (4) + 27 (3)	30 × 27, 118 × 73
*P*. stjordalensis ♀	4	1575 × 1391	230 × 429	63–318 × 208–124–317 × 92–160	29 (2) + 30 (3)	
*P*. *imminuta* s. lat. ♂	3	975 × 867	223 × 338	55–260 × 162–123–272 × 83–138	24 (2) + 24 (2)	53 × 28, 139 × 86
*P*. *imminuta* s. lat. ♀	1	1000 × 900	180 × 410	55–241 × 163–113–280 × 70–148	23 + 24	
*P. longipalpis* ♀	10	2982 × 2870	400 × 717	93–501 × 269–195–587 × 140–253	96 (18) + 97 (14)	
*P. pusilla* ♀	7	805 × 702	194 × 261	28–148 × 108–74–148 × 36–74	17 (3) + 17 (2)	
*P*. sp. A nr *pusilla* ♀	1	730 × 620	175 × 230	28–150 × 100–75–145 × 40–70	22, 23	
*P*. sp. A nr *pusilla* ♂	1	625 × 550	190 × 175	25–110 × 87–55–110 × 30–55	16, 22	53 × 0, 100 × 35
*P*. sp. B nr *pusilla* ♀	1	790 × 690	220 × 260	20–155 × 125–90–175 × 40–80	23, 26	
*P*. *rotundoides* ♀	3	887 × 787	320 × 248	32–223 × 157–109–237 × 54–110	35 (1) + 33 (3)	
*P. rotundoides* ♂	1	770 × 680	300 × 255	35–205 × 150–103–225 × 50–93	38, 36	68 × 0, 158 × 45
*P. variabilis*	9	1047 × 789	135 × 225	30–125 × 83–65–122 × 35–59	9 (1) + 8 (1)	
*P. dispersa*	10	1095 × 921	152 × 258	32–150 × 107–75–141 × 46–69	10 (2) + 11 (1)	

## Results

### Molecular analysis

Cytochrome c oxidase subunit I yielded a 658 bp sequence with 305 variable characters, of which 281 were parsimony informative, the vast majority in third codon positions (Table 3). Region D2 of nuclear ribosomal 28S gave a sequence length of 517–609 bp with 395 variable characters, of which 374 were parsimony informative (Table 3).

**Table 3:** Information on the DNA datasets of *Unionicola* (U), *Piona* (P) and the combined dataset (U + P). Number of base pairs (BP), parsimony informative sites (PI), variable sites (V), constant sites (C), and the percentage of AT base pairs (AT%), for the COI and 28S sequences. The COI sequence is divided into first, second, and third codon positions

Taxa	Gen	Codon	BP	PI	V	C	AT%
U + P	COI	1	210–218	57	67	151	56
		2	211–219	10	21	198	58
		3	210–219	213	216	3	74
	28S		227–609	374	395	336	57
U	COI	1	210–218	24	29	189	56
		2	211–219	1	3	216	59
		3	210–219	167	169	50	70
	28S		536–551	43	49	502	57
P	COI	1	211–218	41	46	172	56
		2	212–219	6	7	212	58
		3	212–219	198	206	13	76
	28S		227–609	312	319	404	57

**Figure 2:**
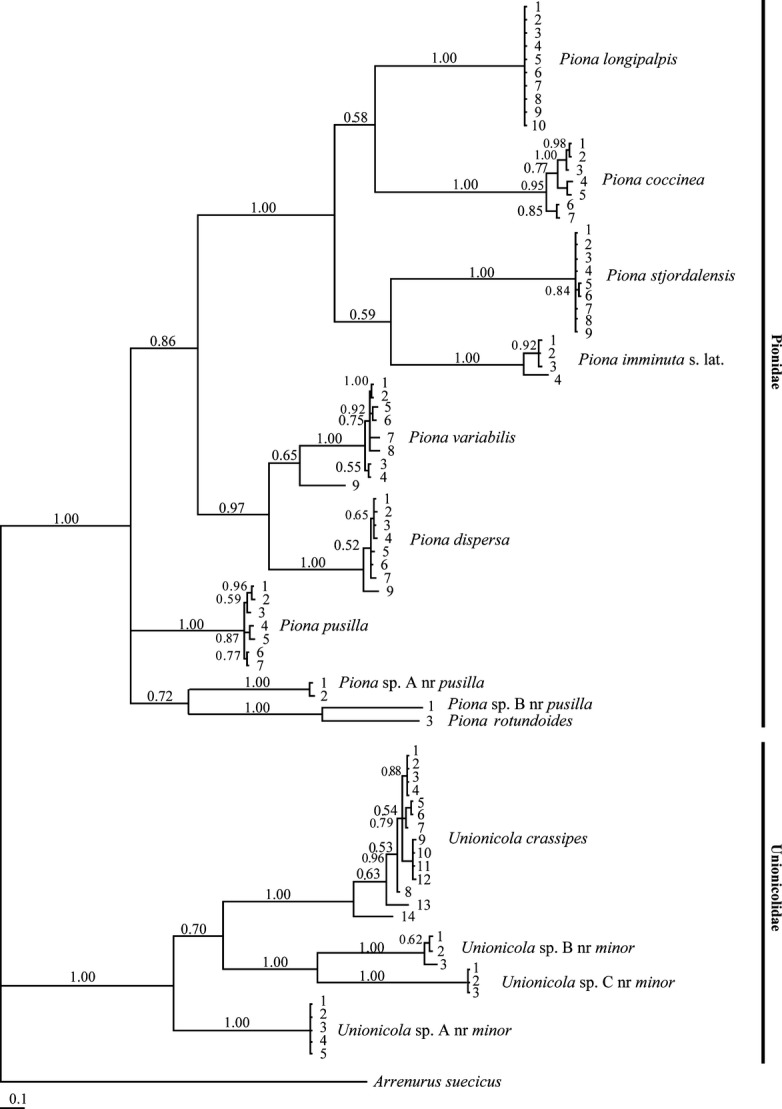
Majority-rule consensus from the Bayesian phylogenetic analysis of COI. Numbers above branches are posterior probability values. Note the large genetic distances between the species (scale bar). Outgroup taxon is *Arrenurus suecicus*.

**Figure 3:**
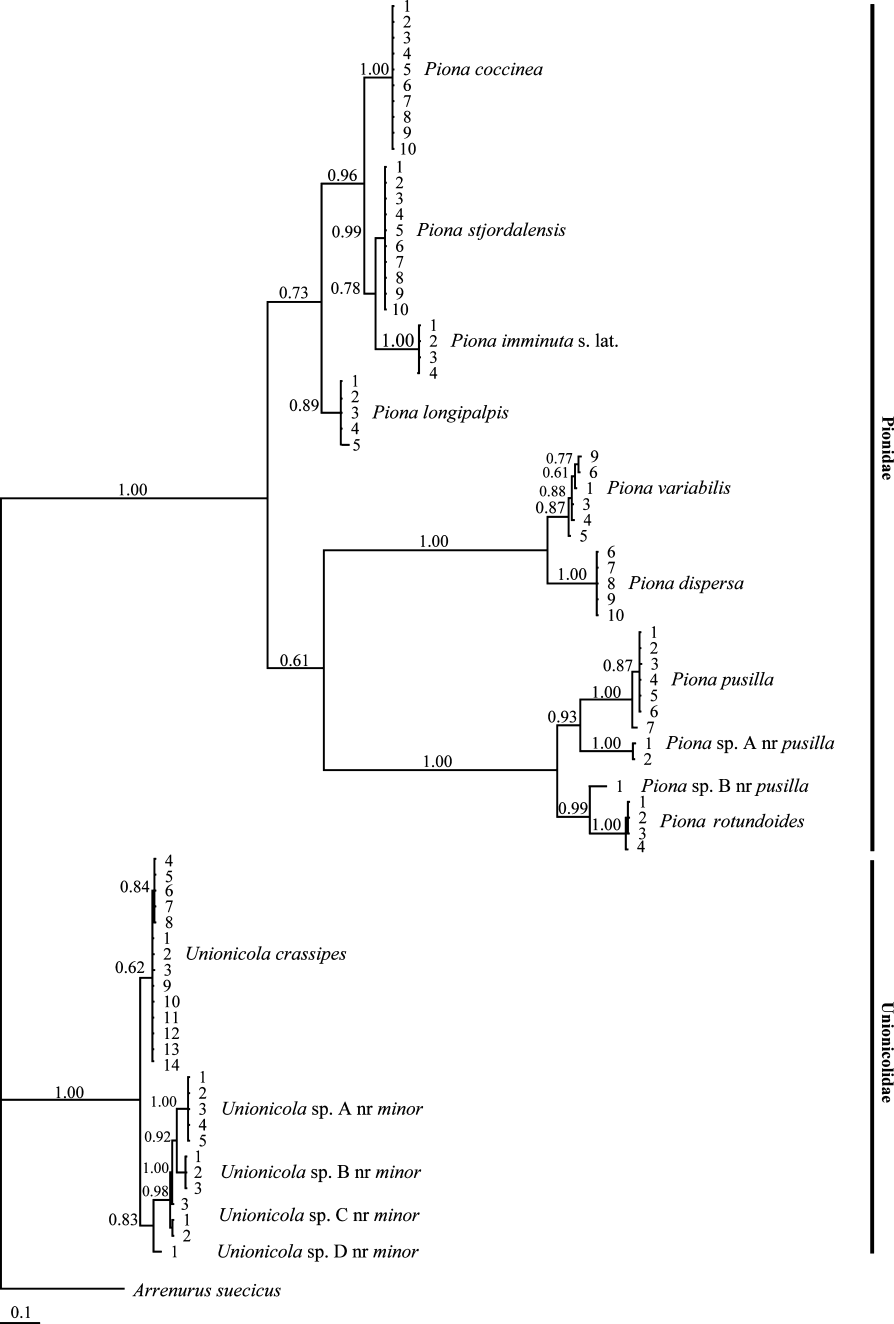
Majority-rule consensus from the Bayesian phylogenetic analysis of 28S. Numbers above branches are posterior probability values. Note the large genetic distances between the species (scale bar). Outgroup taxon is *Arrenurus suecicus*.

Bayesian phylogenetic analyses of COI and 28S gave similar topologies and strong support for most clades, including the two genera *Piona* and *Unionicola* (Figs 2, 3). The main area of disagreement is the relationship between the three complexes of *P. coccinea, P. variabilis,* and *P. pusilla*, where the two genes indicated different solutions, but with weak support. Also within the *P*. *coccinea*-complex, the phylogenetic signals from the two genes conflicted with respect to the relationships among the four specimen clusters, but again without convincing support. The combined Bayesian analysis of COI and 28S lent stronger support to some internal nodes and suggested that *P*. *longipalpis* is the sister species to the *P*. *coccinea*-complex (not shown).

All the examined taxa were separated by large genetic distances from their respective nominate species, presented as COI distances below if not otherwise stated. In addition, some species consisted of more than one distinct genetic cluster. Both genes indicated that *Unionicola crassipes* and *U. minor* together represent a complex of four to five genetic clusters. The 28S tree divided *U. minor* into four clusters (A–D), three of which were matched by COI (COI data were missing for representatives of the fourth cluster, cluster D). A genetic distance of 18% separated *U*. *crassipes* from *U. minor* and clusters A–C were separated with a similar distance from each other (17–23%). The intraspecific distance of *U*. *crassipes* was 6.9% (4.2% without specimen number 14), whereas the average distances within clusters A–C were 0.5% (SD = 0.9). *Piona stjordalensis* and *P. imminuta* s. lat. each formed a distinct cluster with a genetic distance of 19% from each other and separated from *P*. *coccinea* by 21%. One specimen of *P. variabilis* (number 9) differed by 8% in COI from other *P. variabilis* specimens, but this divergence was not reflected in 28S. *Piona variabilis* was separated from *P. dispersa* with a genetic distance of 11%. The intraspecific distance of *P. dispersa* was 1.6%. *Piona pusilla* was composed of three entities ranging from 18% to 27% in genetic distance from each other, in addition to *P. rotundoides* at a similar distance (15–20%). Average differences between nominate species and former forms of *Piona* were 19% (SD = 3.8) compared to 2.6% (SD = 3.0) within clusters. Excluding *P. variabilis* specimen 9, the average intraspecific distance reduced to 1.5% (SD = 1.3). In comparison, the distance of the reference species *Piona longipalpis* (without any described forms) to the congeneric *Piona* species in this study showed a similar range (18–29%), while the 10 sequenced specimens showed no, or very little, intraspecific variation (0% COI, 0–1% 28S).

**Figure 4:**
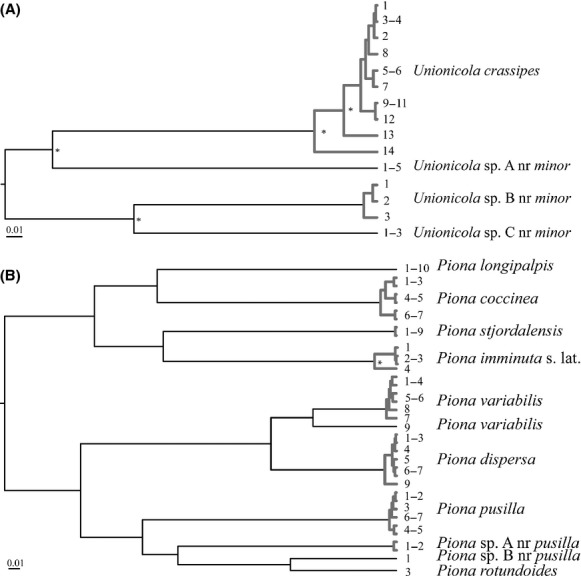
Result from the GMYC species delimitation analyses of the (A) *Unionicola crassipes*-complex and (B) *Piona*-complexes. Light grey lines indicate within-species branches and black lines represent between-species branches. Alternative entities within the ±2Log Likelihood confidence interval are indicated with an asterisk (*).

The favoured GMYC model had a significantly better fit than the null model for the *Piona* species (logL = 196.29 vs. 180.15, df = 2, *P*-value = 9.8 × 10^−8)^ and for the *Unionicola crassipes*-complex (logL = 63.36 vs. 60.20, df = 2, *P*-value = 0.04). Two to six clusters were included in the confidence interval for *Unionicola*, but the maximum likelihood solution was four separate units (*U. crassipes* and the *U. minor* clusters A-C: Fig. 4). For *Piona*, the maximum likelihood model identified 11 separately coalescing mtDNA entities with only one alternative solution included in the confidence interval (marked with * in Fig. 4). In the *P*. *coccinea*-complex, both *P. stjordalensis* and *P. imminuta* s. lat. were confirmed as separately evolving species. *Piona dispersa* was also identified by the model as a distinct species, separate from *P. variabilis*. Finally, *P. pusilla* consisted of three distinct lineages in addition to *P. rotundoides* which was also resolved as a separate coalescing unit. Also specimen number 9 of *P*. *variabilis* constituted a distinct unit from remaining *P. variabilis* specimens, but, as mentioned above, this deep divergence was not reflected by 28S (Fig. 3). The combined GMYC analysis with both *Unionicola* and *Piona* in a common ultrametric tree gave the same results for *Piona,* but the *U*. *crassipes*-complex had a narrower confidence interval, ranging from 4 to 6 separately coalescing units (not shown).

Rosenberg's test is based on sampling individuals from *predefined groups* and testing the probability of reciprocal monophyly given the sample sizes and assuming a single-panmictic population. Two taxa, *Unionicola minor* and *Piona pusilla*, were not monophyletic in one or both of the gene trees in the sense of the *a priori* defined hypothesis as additional genetic clusters were discovered. Likewise, Rodrigo et al. confessed that their branch-length ratio test is too liberal if the hypothesis is defined *a posteriori*. Therefore, we only applied these tests to the hypotheses concerning the remaining *a priori* defined taxa. Rodrigo et al.'s test rejected the null hypothesis for all taxon nodes in the *coccinea* - complex; *P. coccinea*, *P. stjordalensis* and *P. imminuta* s. lat. (*P* < 0.05 for all three). Rosenberg's test of reciprocal monophyly applies to pairs and the null hypothesis could be rejected for both *P. coccinea* versus *P. stjordalensis* + *P. imminuta* s. lat. (*P* = 6.4 × 10^−6^), and for the latter two only (*P* = 2.3 × 10^−4^). The null hypothesis for the reciprocal monophyly of *P. variabilis* and *P. dispersa* was also rejected by Rosenberg's test (*P* = 5.1 × 10^−6^). With Rodrigo et al.'s test, the null hypothesis could be rejected for the *P. dispersa* defining node (*P* < 0.05), but because of specimen no. 9 it could not be rejected for the *P. variabilis* defining node (*P* = 0.3).

### Morphometric analysis

**Figure 5:**
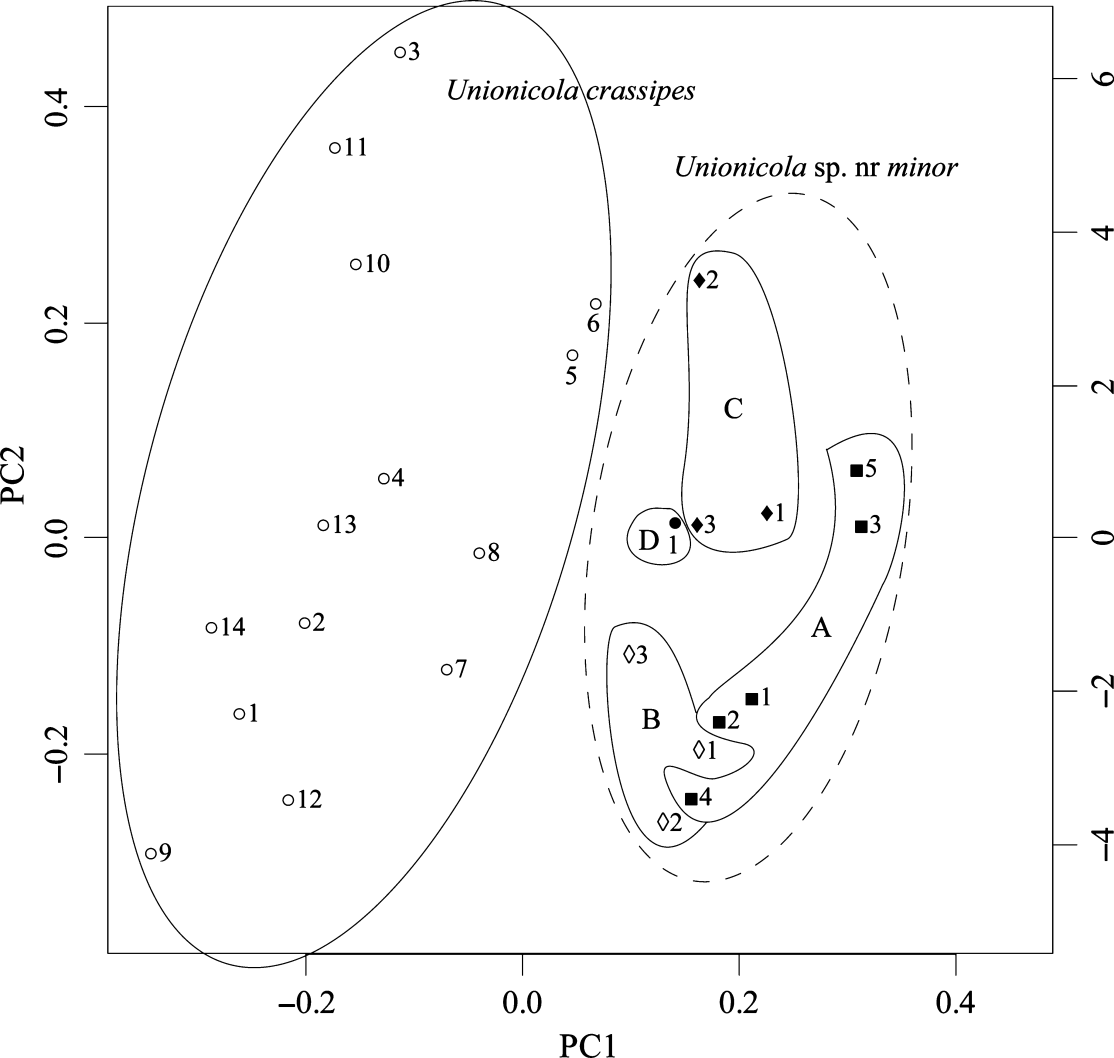
Multivariate analysis on measured morphological characters from *Unionicola* females. Parameters included: dorsal length of palp segments (P-I, P-II, P-III, P-IV, P-V), width of second and fourth segment (P-II, P-IV), body size, and coxa size (length of III+IV and width of IV).

**Figure 6:**
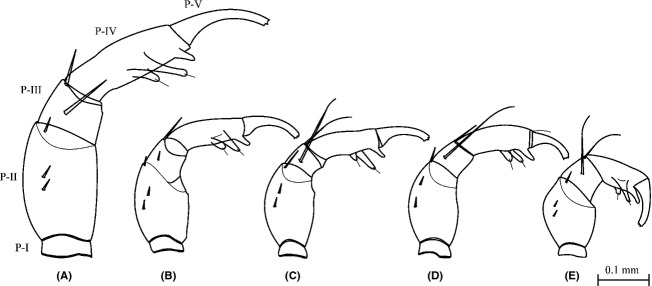
Palp morphology drawn to scale of (A) *Unionicola crassipes* (specimen 1, site 2), (B) *U.* sp. D nr *minor* (specimen 1, site 5), (C) *U.* sp. C nr *minor* (specimen 3, site 5), (D) *U.* sp. B nr *minor* (specimen 3, site 5) and (E) *U.* sp. A nr *minor* (specimen 1, site 4). There is a size difference between *U*. *crassipes* and *U*. *minor* and the ventral side of the second palpal femur (P-II) shows a gradient from straight to convex.

**Figure 7:**
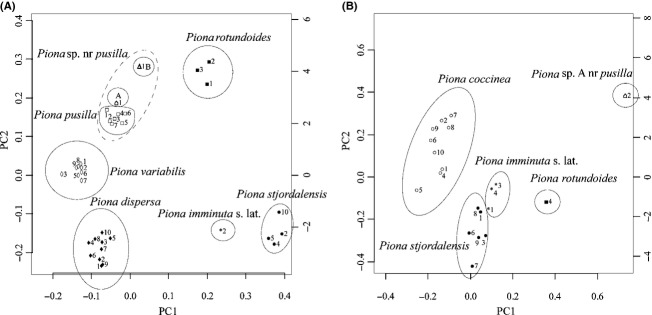
Multivariate analysis on measured morphological characters from *Piona* (A) females and (B) males. Parameters included: dorsal length of palp segments (P-I, P-II, P-III, P-IV, P-V), width of second and fourth segment (P-II, P-IV), coxa of IV (length and width), number of genital acetabula and in females sclerotization (percentage of genital acetabula) and in males length of tarsus and claw of the third leg.

**Figure 8:**
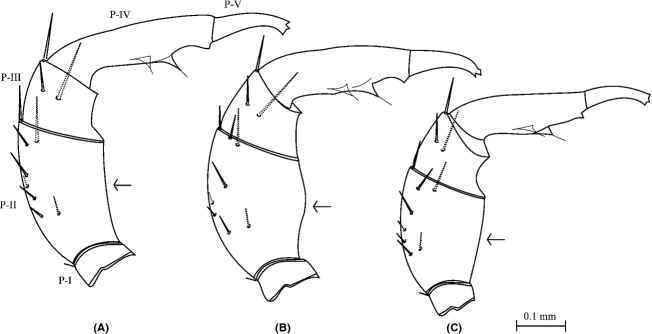
Palp morphology drawn to scale of (A) *Piona coccinea* (specimen 6), (B) *P*. *stjordalensis* (specimen 9) and (C) *P*. *imminuta* s. lat. (specimen 1) of the Lake Mälaren population (site 1 and 2). Differences occur in the ventral side of the second palpal femur (arrow); (A) concave, (B) convex, and (C) straight.

**Figure 9:**
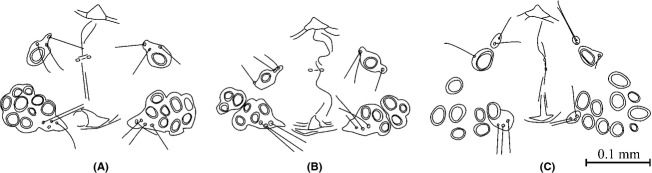
Sclerotization difference of genital acetabula of (A) *Piona varabilis* (specimen 1) (B) *Piona varabilis* specimen 9, and (C) *P. dispersa* (specimen 4). Notice the divided plates on each side of the genital opening in B.

In the morphometric analysis, the first principal component (PC1) represented an isometric size component, whereas the second principal component (PC2) represented shape changes not related to size. For *Unionicola*, only the isometric size component separated *U*. *crassipes* and *U. minor*; *U. crassipes* specimens differ mostly from *U.* sp. A-D nr *minor* due to their larger size (Fig. 5, Table S1). *Unionicola* sp. A–B near *minor* versus C–D near *minor* seems to be partly separable by the second principal component (Fig. 5). High values on the second principle component represent longer and more slender palpal segments relative to the body (Fig. 6, Table S1). *Piona pusilla* and *P. rotundoides* aggregated into distinct clusters mainly along the size axis of PC1 (Fig. 7, Table S1). Along the PC2 axis, *P*. sp. B nr *pusilla* was most similar to *P. rotundoides* due to the higher number of genital acetabula (23+26) (Fig. 7, Table S1). The female of *P*. sp. A nr *pusilla* had 22+23 genital acetabula in contrast to *P. pusilla*, which had an average of 17 acetabula (Table 2). Both the first size component and the second component, reflecting length and width of terminal leg claw, palpal segment width, and the number of genital acetabula, distinguished males of *P. stjordalensis* and *P. imminuta* s. lat. from *P. coccinea* (Figs 7, 8, Table S1). *Piona stjordalensis* and *P*. *imminuta* s. lat. differed mostly along the size component axis of PC1 in both males and females (Fig. 7, Table S1). In the analysis based on females, *P. variabilis* and *P. dispersa* were separated into two distinct clusters based on PC2 representing the number and sclerotization percentage of genital acetabula, the relative length of coxa and the width of palpal segment IV (Fig. 7, Table S1). In general, *P. variabilis* have sclerotized genital plates, whereas *P. dispersa* have no sclerotization, but there is some morphological variation among specimens in this character. Specifically, the examined specimens of *P. dispersa* had between zero and eight sclerotized acetabula, whereas specimens of *P. variabilis* could have incomplete sclerotization displayed as divided plates or one unsclerotized acetabulum (Fig. 9). The reference species, *Piona longipalpis,* had a comparatively large variation in the number of genital acetabula, in contrast to the low genetic variation (Table 2).

## Discussion

### Species delimitation

The Bayesian phylogenetic analyses and the species delimitation with a single locus (e.g., the GMYC model, Rosenberg's and Rodrigo's test) revealed large and consistent genetic distances between all forms with or without already known species status (*U. minor* in relation to *U. crassipes*, *P. stjordalensis* and *P. imminuta* s. lat. both in relation to *Piona coccinea*, *P. rotundoides* in relation to *P. pusilla*, *P. dispersa* in relation to *P. variabilis*). The molecular patterns observed cannot be due to random coalescence processes, but in fact, as they occur sympatrically even in the same locality, support species status with no or limited geneflow between them. Therefore, these taxa cannot be treated as intraspecific variation. The genetic distance between *P. variabilis* and *P. dispera* were lower, but still comparable to the interspecific distances of the examined species, including the reference species of *P. longipalpis*. In fact, the genetic distances in the barcode region were larger than the distances among the majority of recognized, closely related species of other animal groups tested to date (Grant and Bowen 1998 [fish]; Hebert et al. 2003 [moths]; Hebert et al. 2004 [birds]; Hogg and Hebert 2004 [springtails]; Kumar et al. 2007 [mosquitoes]; Koch 2010 [bees]). The differentiation (11–27% including examined species) is, however, similar to closely related species in other groups of Acari (Navajas et al. 1998; Dabert et al. 2008; Skoracka and Dabert 2010; Lv et al. 2013) including water mites (Martin et al. 2010 [18–31%]; Pešić et al. 2012 [11%]).

To use a threshold of genetic distance to identify samples or even to delimit species, for example 2%, has been proposed and used widely, especially in the DNA barcoding literature (Hebert et al. 2003; Hebert et al. 2004; Kumar et al. 2007). However, such a threshold is artificial and not justified by known biological processes. The GMYC is also a method that is based on a simplified threshold and assumes species monophyly, but the value of the threshold is not artificially constructed, but optimized in a maximum likelihood framework based on realistic and established models of intraspecific coalescence and interspecific speciation. Originally developed for species delimitation of community samples in poorly studied groups (Pons et al. 2006), the GMYC model optimizes the transition between a slow interspecific branching rate compared to a relatively faster intraspecific coalescence rate in an ultrametric tree. The combined GMYC likelihood is tested against the likelihood of modeling the entire ultrametric tree as a single coalescence. This test is moderately informative when multiple species in a tree are tested at the same time. In the case of *Piona,* for example, rejecting the null only means that *at least one* of all jointly tested species should be regarded as a separately evolving unit. On the other hand, the ability, or statistical power, to identify the transition in branching rate is reduced if the tested ultrametric tree was to be subdivided into pairs of taxa. Instead, the strength with the GMYC method lies in not requiring an *a priori* species hypothesis and by using a proximate confidence interval of 2 log likelihood units from the maximum likelihood solution (Pons et al. 2006), initial species hypotheses can be erected for further testing beyond the single locus (see also Powell (2012) for an alternative confidence measure). The tests by Rosenberg (2007) and Rodrigo et al. (2008) are better suited to the testing of specific questions as oppose to large-scale biodiversity assessments, and require *a priori* defined hypotheses to be stringent tests (Rodrigo et al. 2008). As putative cryptic species are often discovered as a result of genetic analyses (not *a priori*), for example, in DNA barcoding studies, a careless usage of the one-click plug-in tool applying these tests (Masters et al. 2011) risk unjustified taxonomic inflation. Species delimitation method development is a vibrant and exciting research field where empiricists need to be aware of both pitfalls and potentials.

Despite the molecular support, it is important to not rely solely on a maternally inherited mitochondrial marker when testing species hypotheses, but to corroborate a hypothesis with multiple lines of evidence (de Queiroz 2007). Not the least because a number of potential pitfalls exist, including numts, (Moulton et al. 2010), *Wolbachia* infestation (Whitworth et al. 2007), introgressive hybridization (Sota et al. 2001), incomplete lineage sorting (Funk and Omland 2003), contamination in the lab, and more. In our case, except for specimen 9 of *Piona variabilis*, nuclear 28S is perfectly congruent with all of the COI-defined clusters. What specimen 9 of *P. variabilis* represents is uncertain, but variation in mitochondrial DNA not shown in nuclear or morphological data has been reported in other Acari groups (Leo et al. 2010). It highlights the need to corroborate hypotheses based on mitochondrial markers with nuclear loci and morphology. The quantitative morphometric analysis supported all of the genetically defined species with a combination of morphological characters, except for the challenge represented by the new genetic clusters discovered in the *P*. *pusilla* and *Unionicola crassipes*-complexes. The variation in morphological characters and occurrence of intermediate specimens in these two groups are at the moment problematic for nonmolecular identification. The newly discovered and unnamed genetic clusters aside, the focus of the project was to test if form with or without already known taxonomical status were all supported as valid species by both COI and 28S. Even though there are morphological differences, numerous forms of water mites could not be recognized as species according to Lundblad (1920, 1924, 1962) due to the occurrence of intermediate specimen. Some of the characters( variation between individuals (and occasionally within the same specimen) were congruently observed in this study, but not shown by the genetic data.

### Is *Unionicola minor* a species or a species complex?

Many authors have expressed difficulty in classifying *Unionicola crassipes*-like specimens (Lundblad 1962; Conroy 1979, 1984; Crowell 1984). At present, *U. minor* is a valid species on Fauna Europaea (http://www.faunaeur.org) with the taxonomical comment that it is proposed as subspecies to *U. crassipes* (Gerecke 2011). Even though there are studies on life history, sexual biology, and morphological differences in all life stages providing evidence for two separate species (Hevers 1975, 1977, 1978, 1979a,b, 1980), there are alternative views (Conroy 1979, 1984). Conroy (1979) suggested first that *U. minor* should be discarded and synonymous with *U. crassipes*. However, after reexamination of North American *U. laurentiana* Crowell and Davids 1979 and *U. nearctica* Crowell and Davids 1979; Conroy (1984) concluded that the species complex can be represented by three subspecies; *U. crassipes, U. minor,* and *U. laurentiana*, despite the fact that the first two taxa occur sympatrically (Lundblad 1962; Boyaci and Özkan 2004). As the name implies, *U. minor* is smaller in size and were before 1972 treated only as a form. Our morphological analysis separated *U. crassipes* from *U. minor*, but no further divisions of *U. minor* was obvious. The GMYC analysis, however, gave multiple species delimitation alternatives (2–6 species), and large genetic variation indicated a complex of morphologically very similar species near *U. minor*. This exposes the sensitivity of the GMYC method to taxon sampling. The GMYC method has become quite popular and used in a number of studies (Monaghan et al. 2009; Fontaneto et al. 2011; Isambert et al. 2011), but a note of caution is warranted with regards to the effect of sampling on the outcome. This has only been explored so far with respect to sampling of populations within a species (Lohse 2009; Papadopoulou et al. 2009), but not with respect to the sampling of interspecific variation (see Fujisawa and Barraclough 2013). Analyzed separately, the GMYC confidence interval for *Unionicola* included solutions with between two and six separate units. When analyzed together with the *Piona* dataset, however, the confidence interval only included solutions with four to six units (not shown). However, the maximum likelihood solution in both cases was four units. Including the result of 28S, the genetic analyses indicated a complex of five species: *U. crassipes, U*. sp A nr *minor, U*. sp B nr *minor, U*. sp C nr *minor,* and *U*. sp D nr *minor*. This, together with an overlap in body size, creates uncertainty as to which of the *U. minor* clusters is the most suitable representative of the original description. Therefore, we treat all the clusters as “near *U. minor*”.

It has been implied that size is not a suitable criterion to distinguish species because it might be influenced by environmental factors like nutrition during larval stages (Lundblad 1962; Conroy 1984). The size of the *Unionicola minor* specimens, we studied, does not exactly match the size delimitation of 945 by 734 μm postulated by Viets (1936) and overlaps with the smallest *U. crassipes* (Table 2). However, size differences may contribute to niche separation by affecting the selection of prey (Davids et al. 1981, 1985). *Unionicola crassipes* specimens are larger than *U. minor*, and are therefore able to select larger copepods as prey. Studies on the biology of *U. crasspies* (or *crassipes*-like species), indicate that nymphs and adults in both species prey on small crustaceans and are sponge-associated, while the larvae parasitize flying Chironomidae (Crowell and Davids 1979; Proctor and Pritchard 1989). Furthermore, previous studies have indicated that *U. crassipes* could be genetically isolated from *U. minor* due to the time between the appearance of the first-generation specimens in spring and that the males only deposit spermatophores in the presence of a conspecific female (Hevers 1978; Davids et al. 1985). Except body size, it is important to note the characteristic palpal femur (Lundblad 1962). In the literature, it is stated that *U. crassipes* has a straight palpal femur, while the femur is convex in *U. minor* (Lundblad 1962). However, the palpal femur of the genetically defined species examined here shows a more or less continuous gradient from a straight to a convex profile (Fig. 6). Based primarily on the genetic data, which very clearly separate *U*. *crassipes* and *U*. *minor*, we confirm *U*. *minor* as valid species. However, it is likely that *U*. *minor* in fact is composed of a minimum of four species, as judged from our restricted sample of specimens. Further studies on the *U. minor*-complex, with material from a wider geographic area, are needed to fully elucidate the delimitation and diagnostics of the species in this complex.

### Confirming species statuses in *Piona*

Numerous species in the large family Pionidae, as well as in other water mite families, are rich in variation (Viets 1936; Lundblad 1962; European Water Mite Research 2009; van Haaren and Tempelman 2009). The investigation of Davids and Kouwets's (1987) is the reason why several former varieties are seen as separate species in recent work (Gerecke 2011). They (1987) added morphological characters including larval morphology as an important factor, and these conclusions have also been corroborated by others (Biesiadka 1977; see Viets 1987). Earlier they were each treated as a form or as in *Piona stjordalensis* divided up into multiple taxa (Koenike 1920; Viets 1987). *Piona stjordalensis* was first described as a separate species (Thor 1897). Specialists have later seen it as a form of *P. coccinea* (Sokolow 1940; Láska 1954; Lundblad 1962). The additional forms *P. coccinea f. confertipora* and *P. coccinea f. hankensis* were treated as synonyms to *P. stjordalensis* (Lundblad 1962). Davids and Kouwets (1987) suggested raising *P. stjordalensis* to species level. Furthermore, they contested the opinion by Lundblad (1962) regarding *P. coccinea f. confertipora*. In fact, they synonymized the form, together with *Piona coccinea f. recurva* and *P. coccinea f. gracilipalpis* with *P. imminuta*, due to similar shape of palpal femur. The *P. imminuta* specimens in this study were therefore classified as “*P. imminuta* s. lat.” using the wide concept of *imminuta* sensu Davids and Kouwets (1987) (i.e., including *confertipora*, *recurva* and *gracilipalpis*). *Piona rotundoides* was treated as a form by Lundblad in 1956 and Thor (1897) commented already in the original description that the new species might be a variant or subspecies of *P. pusilla*. However, it is very clear that Davids and Kouwets (1987) were right in their conclusion that *Piona rotundoides* is a valid species, as confirmed by our study.

Regarding identification, we want to point out that the characteristic diagnostic feature of two small papillae on the palpal tibia (P-IV) on *Piona imminuta* s. lat. stated by Piersig (1897) were only present in two females and one of which clearly belonged to another species (*P. stjordalensis* specimen 2). Other authors have noticed that the presence or absence of these papillae vary (Lundblad 1962; Davids and Kouwets 1987). However, a better character seems to be the straight ventral side of the palpal femur (Fig. 8). Davids and Kouwets (1987) mention a smaller palp size in comparison with *P. coccinea* and *P*. *stjordalensis*, but we found no distinct difference in this study. The distinction of *P. coccinea*, *P*. *stjordalensis,* and *P. imminuta* s. lat. were very clear due to shape difference of the tarsus and claws of the males( third leg and palpal femur in both males and females. While *P. coccinea* have a red colour, *P*. *stjordalensis* and *P. imminuta* s. lat. are much paler.

Another taxonomical problem is the character of the amount of genital acetabula. Several species are distinguished by their count, but the intraspecific variation can be substantial (Viets 1936; Lundblad 1962, 1968). Despite this, the number of genital acetabula of *Piona rotundoides* in this study corresponds well with the original description of 30–40 per genital plate (Thor 1897). Moreover, *P. pusilla* is regarded to have 15–22 genital acebula (Davids and Kouwets 1987), but the females of *P*. sp. A-B nr *pusilla* in this study were slightly over the marginal of 22 acetabula per plate (Table 2). *Piona* sp A-B nr *pusilla* probably also represent two distinct species, although the morphological differences recognized to date are subtle and the sampling quite small. This, together with the results from *U. minor*, indicates that there are more species or species complexes present than previously thought.

### *Piona variabilis* and *Piona dispersa*

Until this study, *Piona dispersa* was treated as a synonym of the nominate species *P. variabilis* (European Water Mite Research 2009; Gerecke 2011). However, there are no detailed taxonomical studies and *P. dispersa* has been irregularly treated as a synonym, as a form or as a species (Lundblad 1962; Böttger and Ullrich 1974; etc. see Viets 1987). Böttger and Ullrich (1974) commented after collecting in Germany that they believed these two taxa are conspecific, and it was originally described as a variety of *P. variabilis* due to unsclerotized genital acetabula and no distinguishing features between males (Sokolow 1926). However, the genetic and morphometric data in this study are unequivocal, showing large distances between *P. variabilis* and *P. dispersa*. The molecular analyses point out that the variation in sclerotization around the genital acetabula is intraspecific, yet not evidence of conspecificity with *P. variabilis* (Lundblad 1962). In contrast to the occurrence of incomplete sclerotization connecting some genital acetabula in *P. dispersa*, the opposite pattern is displayed in *P. variabilis* with single acetabula arranged freely without sclerotization. The divided genital plates shown in specimen 9 of *P. variabilis* (Fig. 9) coincide with the divergent COI sequence (Figs 2, 4). However, we refrain from assigning taxonomic importance to this character because of (1) the lack of genetic differentiation in 28S, (2) the coherence of the *P*. *variabilis* cluster in the morphometric PCA analysis, and (3) the same feature detected on two other specimens, but on only one side of the genital opening (a total of three plates). Despite the intraspecific and overlapping variation, this character alone can well be used to identify *P. dispersa*. We imagine the two species can coexist, perhaps due to small differences in prey selection, behaviour or habitat preference which often explains species coexistance (Davids et al. 1981). Some 30–40 species of over 5000 parasitengonine mites are recorded to have a loss of larval parasitism, including the *P. coccinea* and the *P. pusilla* group (Smith 1998). This could also explain how two closely related species can occur sympatrically, one with typical parasitic larvae and the other with nonfeeding larvae (Smith 1998).

### Taxonomic changes

Following our results with both molecular and morphological data, *Piona dispersa* is a valid species, separated from the nominate species *P. variabilis*. We propose that *P. dispersa* is raised to species level. Our results also show that *Unionicola minor* and *P*. *pusilla* consist of at least three cryptic species each, which we refrain from formally naming here in the anticipation of future morphological studies uncovering reliable diagnostic characters separating these species.

## Conclusions

Species statuses as suggested by Davids and Kouwets (1987) based on morphological comparison of *Piona imminuta s. lat., P. stjordalensis,* and *P. rotundoides,* are now strongly supported with both molecular and morphometric analyses with this study. Likewise, the new species level status of *P. dispersa* is established with the same criteria. We make the assumption that more diversity is present in water mites than thought in the past, not only uncovered when described “forms” are shown to be valid species but also through molecular data revealing cryptic species complexes. Even if our study clarifies only a fragment of this really problematic topic, it has demonstrated the utility of explicit species delimitation methods to test taxonomic questions at the species-to-population level. The sympatric occurrence, a convergence toward a unified species concept (de Queiroz 2007) and implementation of the coalescent process model as a null hypothesis are key elements to species delimitation for the future.

## Appendix 1 Details of the 94 specimens included in the study. Vouchers have the collection numbers NHRS-ACAR 000000001-94, and are deposited at the Swedish Museum of Natural History (NHRS). In the table ‘F’ stands for female and ‘M’ for male specimen. Localities (site 1–6) are described in Figure 1 and Table 1. Each specimen has a Morphbank accession number and each sequence a Genbank accession number

**Table:**

					Genbank		
						
Specimen	Sex	Voucher	Site Sampled	Site	COI	28S	Morphbank
*Unionicola crassipe-*complex							
*Unionicola crassipes*							
1	F	NHRS-ACAR 000000001	2008-06-17	2	JN034803	JN034883	647460
2	F	NHRS-ACAR 000000002	2008-06-17	2	JN034802	JN034882	647461
3	F	NHRS-ACAR 000000003	2008-07-22	5	JN034801	JN034881	647462
4	F	NHRS-ACAR 000000004	2008-06-17	2	JN034800	JN034880	647463
5	F	NHRS-ACAR 000000005	2008-07-22	5	JN034799	JN034879	647464
6	F	NHRS-ACAR 000000006	2008-07-22	5	JN034798	JN034878	647465
7	F	NHRS-ACAR 000000007	2008-06-17	2	JN034797	JN034877	647466
8	F	NHRS-ACAR 000000008	2008-06-17	2	JN034796	JN034876	647467
9	F	NHRS-ACAR 000000009	2008-06-17	2	JN034795	JN034875	647468
10	F	NHRS-ACAR 000000010	2008-04-05	4	JN034794	JN034874	647470
11	F	NHRS-ACAR 000000011	2008-06-17	2	JN034793	JN034873	647471
12	F	NHRS-ACAR 000000012	2008-06-17	2	JN034792	JN034872	647472
13	F	NHRS-ACAR 000000013	2008-06-17	2	JN034791	JN034871	647473
14	F	NHRS-ACAR 000000014	2008-06-17	2	JN034790	JN034870	647474
*Unionicola* sp. A nr *minor*							
1	F	NHRS-ACAR 000000015	2008-04-05	4	JN034814	JN034895	647475
2	F	NHRS-ACAR 000000016	2008-04-05	4	JN034813	JN034894	647476
3	F	NHRS-ACAR 000000017	2008-04-05	4	JN034812	JN034893	647477
4	F	NHRS-ACAR 000000018	2008-04-05	4	JN034811	JN034892	647478
5	F	NHRS-ACAR 000000019	2008-04-05	4	JN034810	JN034891	647479
*Unionicola* sp. B nr *minor*							
1	F	NHRS-ACAR 000000020	2008-06-17	2	JN034809	JN034890	647480
2	F	NHRS-ACAR 000000021	2008-06-17	2	JN034808	JN034889	647481
3	F	NHRS-ACAR 000000022	2008-07-22	5	JN034807	JN034888	647482
*Unionicola* sp. C nr *minor*							
1	F	NHRS-ACAR 000000023	2008-07-22	5	JN034806	JN034887	647483
2	F	NHRS-ACAR 000000024	2008-07-22	5	JN034805	JN034886	647484
3	F	NHRS-ACAR 000000025	2008-07-22	5	JN034804	JN034885	647485
*Unionicola* sp. D nr *minor*							
1	F	NHRS-ACAR 000000026	2008-07-22	5		JN034884	647486
*Piona coccinea*-complex							
*Piona coccinea*							
1	M	NHRS-ACAR 000000027	2008-06-17	1	JN034738	JN034825	647306
2	M	NHRS-ACAR 000000028	2007-06-01	1	JN034737	JN034824	647307
3	M	NHRS-ACAR 000000029	2007-06-01	1	JN034736	JN034823	647308
4	M	NHRS-ACAR 000000030	2008-06-17	2	JN034735	JN034822	647303
5	M	NHRS-ACAR 000000031	2007-06-01	2	JN034734	JN034821	647304
6	M	NHRS-ACAR 000000032	2008-06-17	2	JN034733	JN034820	647305
7	M	NHRS-ACAR 000000033	2008-06-17	1	JN034732	JN034819	647309
8	M	NHRS-ACAR 000000034	2008-06-17	1		JN034818	647310
9	M	NHRS-ACAR 000000035	2008-06-17	1		JN034817	647311
10	M	NHRS-ACAR 000000036	2008-07-22	5		JN034816	647312
*Piona stjordalensis*							
1	M	NHRS-ACAR 000000037	2008-06-17	1	JN034780	JN034863	647313
2	F	NHRS-ACAR 000000038	2008-06-17	1	JN034779	JN034862	647314
3	M	NHRS-ACAR 000000039	2008-06-17	1	JN034778	JN034861	647315
4	F	NHRS-ACAR 000000040	2008-06-17	1	JN034777	JN034860	647316
5	F	NHRS-ACAR 000000041	2008-06-17	1	JN034776	JN034859	647317
6	M	NHRS-ACAR 000000042	2008-06-17	1	JN034775	JN034858	647318
7	M	NHRS-ACAR 000000043	2008-06-17	1	JN034774	JN034857	647319
8	M	NHRS-ACAR 000000044	2008-06-17	1	JN034773	JN034856	647320
9	M	NHRS-ACAR 000000045	2008-06-17	1	JN034772	JN034855	647321
10	F	NHRS-ACAR 000000046	2008-06-17	1		JN034854	647322
*Piona imminuta* s. lat.							
1	M	NHRS-ACAR 000000047	2008-06-17	1	JN034750	JN034834	647326
2	F	NHRS-ACAR 000000048	2008-07-22	5	JN034749	JN034833	647327
3	M	NHRS-ACAR 000000049	2008-06-17	1	JN034748	JN034832	647328
4	M	NHRS-ACAR 000000050	2008-07-22	5	JN034747	JN034831	647329
*Piona longipalpis*							
1	F	NHRS-ACAR 000000051	2008-06-17	1	JN034760	JN034839	476669
2	F	NHRS-ACAR 000000052	2008-06-17	1	JN034759	JN034838	464445
3	F	NHRS-ACAR 000000053	2008-06-17	2	JN034758	JN034837	476690
4	F	NHRS-ACAR 000000054	2008-06-17	2	JN034757	JN034836	476692
5	F	NHRS-ACAR 000000055	2008-06-17	1	JN034756	JN034835	476673
6	F	NHRS-ACAR 000000056	2008-06-17	1	JN034755		476670
7	F	NHRS-ACAR 000000057	2008-06-17	1	JN034754		476675
8	F	NHRS-ACAR 000000058	2008-06-17	2	JN034753		476678
9	F	NHRS-ACAR 000000059	2008-06-17	2	JN034752		476694
10	F	NHRS-ACAR 000000060	2008-06-17	2	JN034751		476696
*Piona pusilla*-complex							
*Piona* pusilla							
1	F	NHRS-ACAR 000000061	2008-06-17	1	JN034767	JN034846	647435
2	F	NHRS-ACAR 000000062	2008-06-17	1	JN034766	JN034845	647436
3	F	NHRS-ACAR 000000063	2008-06-17	1	JN034765	JN034844	647437
4	F	NHRS-ACAR 000000064	2008-06-17	1	JN034764	JN034843	647438
5	F	NHRS-ACAR 000000065	2008-06-17	1	JN034763	JN034842	647439
6	F	NHRS-ACAR 000000066	2008-06-17	1	JN034762	JN034841	647440
7	F	NHRS-ACAR 000000067	2008-06-17	1	JN034761	JN034840	647441
*Piona* sp. A nr *pusilla*							
1	F	NHRS-ACAR 000000068	2008-07-22	6	JN034771	JN034853	647444
2	M	NHRS-ACAR 000000069	2008-07-22	5	JN034770	JN034852	647445
*Piona* sp. B nr *pusilla*							
1	F	NHRS-ACAR 000000070	2008-06-17	1	JN034769	JN034851	647442
*Piona rotundoides*							
1	F	NHRS-ACAR 000000071	2008-06-17	1		JN034850	647350
2	F	NHRS-ACAR 000000072	2008-07-22	5		JN034849	647351
3	F	NHRS-ACAR 000000073	2008-07-22	5	JN034768	JN034848	647352
4	M	NHRS-ACAR 000000074	2008-06-17	3		JN034847	647354
*Piona variabilis*-complex							
*Piona variabilis*							
1	F	NHRS-ACAR 000000075	2008-06-17	1	JN034789	JN034869	647331
2	F	NHRS-ACAR 000000076	2008-06-17	1	JN034788		647332
3	F	NHRS-ACAR 000000077	2007-06-01	1	JN034787	JN034868	647333
4	F	NHRS-ACAR 000000078	2007-06-01	1	JN034786	JN034867	647334
5	F	NHRS-ACAR 000000079	2007-06-01	1	JN034785	JN034866	647335
6	F	NHRS-ACAR 000000080	2008-06-17	1	JN034784	JN034865	647336
7	F	NHRS-ACAR 000000081	2008-06-17	1	JN034783		647337
8	F	NHRS-ACAR 000000082	2008-06-17	1	JN034782		647338
9	F	NHRS-ACAR 000000083	2008-06-17	1	JN034781	JN034864	647339
*Piona dispersa*							
1	F	NHRS-ACAR 000000084	2007-06-01	1	JN034746		647340
2	F	NHRS-ACAR 000000085	2007-06-01	1	JN034745		647341
3	F	NHRS-ACAR 000000086	2007-06-01	1	JN034744		647342
4	F	NHRS-ACAR 000000087	2007-06-01	1	JN034743		647343
5	F	NHRS-ACAR 000000088	2007-06-01	1	JN034742		647344
6	F	NHRS-ACAR 000000089	2007-06-01	1	JN034741	JN034830	647345
7	F	NHRS-ACAR 000000090	2007-06-01	1	JN034740	JN034829	647346
8	F	NHRS-ACAR 000000091	2008-06-17	1		JN034828	647347
9	F	NHRS-ACAR 000000092	2007-06-01	1	JN034739	JN034827	647348
10	F	NHRS-ACAR 000000093	2008-06-17	1		JN034826	647349
*Arrenurus suecicus*							
1	M	NHRS-ACAR 000000094	2008-06-17	2	JN034731	JN034815	659537

## Supporting Information

**Table S1.** Characters (length = l, width = w) used in the principal component analyses of *Unionicola* and *Piona* and loadings of each character on principal components 1 and 2.
