# Supplementary material for: “Forms” of water mites (Acari: Hydrachnidia): intraspecific variation or valid species?
Source: Ecol Evol. 2013 Aug 28;3(10):3415–35. doi: 10.1002/ece3.704 (PMC3797488; doi:10.1002/ece3.704)
Supplement: Supplementary file 1 [file ece30003-3415-SD1.doc]

SUPPLEMENTARY

Supplementary Table 1. Characters (length=l, width=w) used in the principal component analyses of *Unionicola* and *Piona* and loadings of each character on principal components 1 and 2.

| *Unionicola* females | | | *Piona* females | |  | *Piona* males | |  |
| --- | --- | --- | --- | --- | --- | --- | --- | --- |
| Character | PC1 | PC2 | Character | PC1 | PC2 | Character | PC1 | PC2 |
| Body (l) | -0.295 | -0.398 | Coxa (l) | 0.262 | 0.382 | Coxa (l) | -0.284 | 0.192 |
| Body (w) | -0.281 | -0.573 | Coxa (w) | 0.312 | -0.158 | Coxa (w) | -0.336 | 0.017 |
| Coxa (l) | -0.318 | 0.092 | Genital acebula (left side) | 0.280 | 0.370 | Genital acebula (left side) | 0.004 | -0.351 |
| Coxa (w) | -0.320 | -0.020 | Genital acebula (right side) | 0.288 | 0.324 | Genital acebula (right side) | 0.089 | -0.341 |
| P-I (l) | -0.282 | 0.430 | Sclerotization percentage | 0.109 | 0.556 | P-I (l) | -0.332 | -0.006 |
| P-II (l) | -0.309 | 0.259 | P-I (l) | 0.265 | -0.377 | P-II (l) | -0.305 | -0.209 |
| P-III (l) | -0.311 | 0.056 | P-II (l) | 0.319 | -0.128 | P-III (l) | -0.260 | -0.261 |
| P-IV (l) | -0.319 | 0.240 | P-III (l) | 0.314 | -0.003 | P-IV (l) | -0.329 | -0.122 |
| P-V (l) | -0.316 | 0.270 | P-IV (l) | 0.322 | -0.055 | P-V (l) | -0.336 | -0.056 |
| P-II (w) | -0.295 | -0.141 | P-V (l) | 0.319 | -0.093 | P-II (w) | -0.095 | -0.457 |
| P-IV (w) | -0.263 | -0.315 | P-II (w) | 0.313 | -0.084 | P-IV (w) | -0.195 | -0.359 |
|  |  |  | P-IV (w) | 0.294 | -0.316 | III Leg 6. (l) | -0.256 | 0.190 |
|  |  |  |  |  |  | III Leg 6. (w) | -0.313 | 0.172 |
|  |  |  |  |  |  | Claw (l) | -0.129 | 0.373 |
|  |  |  |  |  |  | Claw (w) | -0.281 | 0.227 |
